# *SamPler* – a novel method for selecting parameters for gene functional annotation routines

**DOI:** 10.1186/s12859-019-3038-4

**Published:** 2019-09-05

**Authors:** Fernando Cruz, Davide Lagoa, João Mendes, Isabel Rocha, Eugénio C. Ferreira, Miguel Rocha, Oscar Dias

**Affiliations:** 1 Centre of Biological Engineering, University of Minho, 4710-057 Braga, Portugal; 2 Instituto de Tecnologia Química e Biológica, Universidade Nova de Lisboa, 2780-157 Oeiras, Portugal

**Keywords:** *SamPler*, Annotation routines, Parametrization, *Merlin*

## Abstract

**Background:**

As genome sequencing projects grow rapidly, the diversity of organisms with recently assembled genome sequences peaks at an unprecedented scale, thereby highlighting the need to make gene functional annotations fast and efficient. However, the (high) quality of such annotations must be guaranteed, as this is the first indicator of the genomic potential of every organism.

Automatic procedures help accelerating the annotation process, though decreasing the confidence and reliability of the outcomes. Manually curating a genome-wide annotation of genes, enzymes and transporter proteins function is a highly time-consuming, tedious and impractical task, even for the most proficient curator. Hence, a semi-automated procedure, which balances the two approaches, will increase the reliability of the annotation, while speeding up the process. In fact, a prior analysis of the annotation algorithm may leverage its performance, by manipulating its parameters, hastening the downstream processing and the manual curation of assigning functions to genes encoding proteins.

**Results:**

Here *SamPler*, a novel strategy to select parameters for gene functional annotation routines is presented. This semi-automated method is based on the manual curation of a randomly selected set of genes/proteins. Then, in a multi-dimensional array, this sample is used to assess the automatic annotations for all possible combinations of the algorithm’s parameters. These assessments allow creating an array of confusion matrices, for which several metrics are calculated (accuracy, precision and negative predictive value) and used to reach optimal values for the parameters.

**Conclusions:**

The potential of this methodology is demonstrated with four genome functional annotations performed in *merlin*, an in-house user-friendly computational framework for genome-scale metabolic annotation and model reconstruction. For that, *SamPler* was implemented as a new plugin for the *merlin* tool.

**Electronic supplementary material:**

The online version of this article (10.1186/s12859-019-3038-4) contains supplementary material, which is available to authorized users.

## Background

The emergence of high-throughput sequencing techniques led to a fast increase in the number of genome sequencing projects over the years, with over 196,000 finished or ongoing projects, as of April 2018 [1]. It is clear that, nowadays, the functional annotation of these genome sequences is eased by automated methodologies and workflows. Several authors have been reporting novel computational tools [2–6] and workflows built essentially as meta-servers [7–15], to automate the annotation of genes encoding proteins. Though the effort of the Gene Ontology (GO) Consortium [16–18] and others [19–23] has been notorious, the emergence of multiple methodologies and platforms to perform genome functional annotation hindered the aim for data unification and spread redundant information across multiple repositories and databases. Surprisingly, multiple authors have reported errors in genes’ functional annotations over the years, pointing out high misannotation levels (up to 80% in some cases) in single-genome annotations [24, 25], single-gene annotations [26], large-scale data repositories [27, 28], or even in the GO database [29, 30]. Concerns about this issue increase when assessing misannotations of molecular functions in the four major public protein sequence databases: Universal Protein Resource Knowledgebase (UniProtKB)/TrEMBL, UniProtKB/Swiss-Prot, GenBank NR [31] and Kyoto Encyclopedia of Genes and Genomes (KEGG) [32]. According to Schnoes and coworkers [33], the manually curated database UniProtKB/Swiss-Prot, shows levels of misannotation close to 0, whereas the three non-curated protein sequences databases (GenBank NR, UniProtKB/TrEMBL and KEGG) exhibit high levels of misannotations averaging 5–63%, and in specific cases up to 80%.

Genome annotation can be divided into structural annotation and functional annotation. Whereas some of the mentioned computational tools and meta-servers can perform both [7–14], this work will focus on a novel method for leveraging genome functional annotations, namely enzyme and transporter protein functional annotation.

Detailed, accurate and useful gene products’ descriptions are regularly sought when retrieving high-level gene functional annotations. Although simple terms, such as dehydrogenase, can describe gene products, accurate and thoroughly refined terms as pyruvate dehydrogenase (NADP+) should be used instead. Additionally, besides systematic database accession numbers and gene products’ descriptions, special identifiers such as the Enzyme Commission (EC) [34] and Transport Classification (TC) numbers [35] are vastly used nowadays to classify enzymatic and transporter proteins, respectively. These special identifiers should, whenever possible, be included in the annotations, to increase the scope and decrease the ambiguity of genome functional annotations.

Most publicly available frameworks for the automation of genome functional annotations are complex pipelines made of multiple processes using several tools and algorithms, based on similarity and/or profile searches [2, 3, 9–11], subsystem-based functional roles [12], domain prediction and annotation [8, 11], genome annotations comparison [5, 6, 15]. These, in turn, are sensitive to parameters, cut-offs and quality assessments.

Cases in which the need to obtain fast gene products’ annotations justifies the use of such frameworks, should also guarantee the quality of the annotation. Interestingly, current frameworks indeed claim to provide both fast and accurate genome annotations. Nevertheless, parameters and cut-off thresholds are rarely changed when submitting data, and descriptions of the methodologies that lead to specific outcomes are seldom available [5, 7–14]. Hence, the annotations of genes encoding proteins may have been inferred from biased and redundant data without user’s awareness.

Genome-wide functional annotations are often performed using similarity search algorithms, such as the Basic Local Alignment Search Tool (BLAST) [36], Smith-Waterman alignments [37] or HMMER [38]. These and other similar algorithms compare sequences with other sequences (typically in sequence databases), providing clusters of genes with similar sequences, which in theory should have similar functions.

Occasionally, functional annotations may be erroneously inferred due to: a biased taxonomic distribution of the homologous genes, which may be systematically reported by alignment algorithms; the presence of incorrectly annotated homologous genes; the systematic presence of homologous genes assigned with unknown functions or hypothetical proteins; and, the spread of redundancy across multiple databases. These problems enhance the need to manually curate gene functional annotations. However, manual curation is often time-consuming and requires a huge effort, even from expert curators.

Cases in which the user is allowed to configure the annotation workflow may benefit from a prior meticulous analysis of the data and a fine tuning of the computational algorithm parameters, which may hasten downstream processing.

Moreover, other authors highlighted the importance of pairing manual curation (namely, inferred from literature) with computational predictions or using multiple databases as information sources, to update genes’ annotations [5, 6, 15, 20, 23, 39].

As far as our knowledge allows, here we provide the only method (*SamPler*) aimed at determining the best settings for the parameters of genome functional annotation algorithms, through the manual annotation of a random sample of entities.

The approach demonstrated in this study is used to determine the best configuration of *merlin*’s [2] enzyme annotation algorithm, setting the α parameter, which leverages two scores (namely the frequency and taxonomy scores), while proposing upper and lower thresholds to automatically accept or reject gene’s metabolic annotations, respectively.

Furthermore, this strategy prevents the utilization of biased and redundant data.

The method was implemented as a new plugin for *merlin*’s current version, and thus made available for the community, making the reproducibility of our results possible with minimum effort. Using the *SamPler* plugin, one can now choose between two gene functional annotation routines available in *merlin*: automatic first-hit annotation or *SamPler* for leveraging the enzyme’s annotation algorithm.

Remarkably, this method can have many other applications besides the one demonstrated herein.

## Methods

### merlin

*merlin* is a user-friendly computational tool which, among other features, allows performing genome-wide metabolic (re-)annotations and the reconstruction of genome-scale metabolic models [2].

*merlin* performs two types of metabolic (re-) annotation, namely the enzymes and transporters annotation. The methodology proposed in this work was implemented as a new plugin that leverages the enzymes annotation algorithm, thereby being fully available for *merlin*’s current version.

In *merlin*, the enzymes annotation involves performing remote similarity searches (either with BLAST or HMMER) and assigning a function to each gene, taking into account the homologous genes functions. Genes encoding enzymes should have at least one homologous gene assigned with an EC number. The likelihood of a gene being assigned with an EC number is determined by an internal scoring algorithm that takes into account both the frequency (cardinality of such EC number among the orthologous genes) and the taxonomy (taxonomic proximity between the organisms for a given EC number), to calculate a score (between 0 and 1). The higher the score, the higher the chance of that gene encoding such enzyme. More information on this algorithm can be found in [2].

The trade-off between the frequency and the taxonomy scores is attained by a parameter, the α value. In previous versions of the framework, the user set this parameter and the challenge of this work is to provide an automatic method to calculate this value, found to be of a great importance in the results of the annotation.

*merlin* also allows performing automatic gene functional annotations. In this case, a first-hit annotation pipeline is followed by using an annotation workflow such as the one described in Additional file 1. Nevertheless, manual curation and other factors influencing the outcome of the annotation such as the frequency of a given annotation is not considered.

### Implementation

Currently, the methodology described in this work, *SamPler*, is implemented and used to select a value for the α parameter, together with the thresholds for accepting annotations and rejecting genes as enzymatic. The proposed method suggests the curation of a random sample of entries from an annotation project, using these to automatically select the parameters for configuring the algorithm that performs automatic enzymes annotation, being integrated in the software *merlin*.

Alternatively, the method can be used to configure any other annotation algorithm or workflow. For this to be possible, these computational tools or meta-servers should return a score or other rank for each entry as a function of the parameters configuration.

#### Selection and annotation of a standard of truth

This process begins by setting an initial sensible value for all parameters and automatically running the enzyme annotation algorithm or workflow. Then, a sample with *x*1 genes (5% to 10% of the genes/proteins to be annotated) should be (randomly) selected, guaranteeing that all possible score intervals are represented and the number of entities in each interval is similar.

These records should be manually curated with a defined workflow (an example of a manual curation workflow for *merlin* is shown in the Additional file 1). These entries will become the *standard of truth* for evaluating the genes functional annotations proposed by the tool.

#### Parameters assessment

**Fig. 1:**
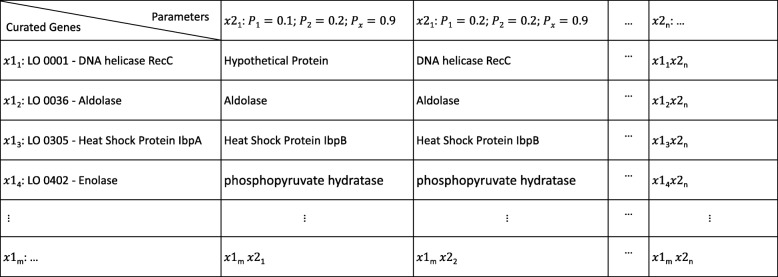
Scheme of a multi-dimensional array (*x*1, *x*2). This scheme is used for evaluating randomly selected annotations (*x*1) automatically proposed by a tool, against annotations curated through a manual curation workflow (standard of truth). The multi-dimensional array allows evaluating all possible combinations of values for the tool’s parameters (*x*2)

This evaluation starts with the creation of a multi-dimensional array, of dimensions (*x*1, *x*2) that features the selected genes on the rows and all possible combinations of parameters (*x*2) on the columns, as shown in Fig. 1.

This array will comprise the framework’s automatic annotations for sample *x*1, for all possible combinations of the algorithm’s parameters (*x*2). These combinations may lead to different automatic annotations that, when compared to the *standard of truth*, allow assessing the best configuration of the parameters’ settings.

This comparison allows creating a new multi-dimensional array of dimensions (*t*, *x*2), which helps simplifying the analysis of the results. Such array will have score thresholds (*t*) between the minimum and the maximum score in the rows, and all possible parameter combinations *x*2 in the columns. Each intersecting cell will contain a confusion matrix for each (*t*, *x*2) pair, as shown in Table S.1 of the Additional file 2.

**Fig. 2:**
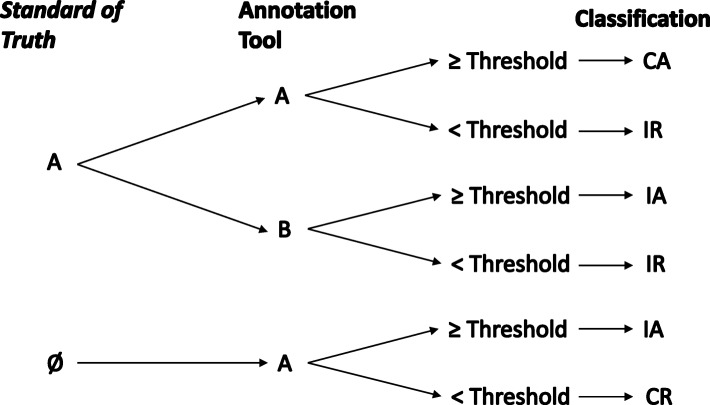
Evaluation of an annotation algorithm when compared with the Standard of Truth (A or Ø), determined with a manual curation workflow. If the manual annotation is equal to the algorithm’s annotation (A = A) and has a score above the threshold (≥ Threshold), it is classified as CA. When the algorithm’s annotation is below the threshold (≤ Threshold), it is classified as IR. Alternatively, when the annotation is different from the manual curation (A ≠ B), the annotation will be classified either as IA or IR, when above (≥ Threshold) or below (≤ Threshold) the threshold, respectively. Finally, the manual curation may indicate that the entity should not be annotated (Ø), whereas the tool assigned an annotation (A). These cases will be classified as IA or CR, whenever the annotation’s score is above or below the threshold, respectively

These confusion matrices evaluate the performance of the different conditions of the algorithm or workflow and report the number of incorrect annotations (IA – similar to false positives), incorrect rejections (IR – similar to false negatives), correct annotations (CA – similar to true positives) and correct rejections (CR – similar to true negatives), according to Fig. 2.

The two former conditions represent type I and type II errors. The first case (IA) may be the outcome of two distinct situations: the algorithm annotation is incorrect (A ≠ B), or the entity should not be annotated (A ≠ Ø), though the algorithm annotation score is higher (or equal) than the provided threshold. The second case (IR) may also arise from two distinct situations: the algorithm’s annotation score is below the threshold, despite being correct (A = A) or even if it is incorrect (A ≠ B).

Finally, the two latter cases represent cases in which automatic annotations, above or equal to the threshold, are in agreement (A = A) with the curated annotation (CA), or entities that should not have been annotated (A ≠ Ø) have annotation scores below the threshold (CR), respectively.

Nevertheless, these rules can be changed and adapted to other situations, depending on the paradigm and objective of the annotation. Different premises regarding the conditions of the algorithm and the curation workflow, will provide different outcomes that will lead to distinct classifications.

Confusion matrices allow calculating several different metrics. For this work, *accuracy*, *precision* and *negative predictive value* ( *NPV*) will be considered. *Accuracy* reveals how often the workflow makes correct annotations and rejections, and is calculated as follows:

1 $$ accuracy=\frac{CA+ CR}{CA+ CR+ IA+ IR} $$

On the other hand, *precision* assesses how often the algorithms assignments are correct, according to:

2 $$ precision=\frac{CA}{CA+ IA} $$

Finally, the *NPV* evaluates how often the algorithm’s rejections are correct:

3 $$ NPV=\frac{CR}{\  CR+ IR} $$

These rates allow selecting the best values for several parameters, together with upper and lower thresholds for the annotations’ scores. The average *accuracy* of each column allows determining which parameters’ settings (*x*2) attain higher accuracy, i.e. which set of values for all parameters yields more correct annotations and rejections. Then, for such column, *precision* and *NPV* are used to determine the upper and lower thresholds, respectively. Every row *t* with a *precision* of 1 indicates that the algorithm’s annotations are always correct above the respective threshold, hence these should not require curation. Likewise, rows with *NPV* of 1 indicate that the annotation algorithm correctly rejects all annotations below such *t*.

Therefore, the upper threshold should be the lowest *t* with the highest *precision* (ideally 1), and the lower threshold the highest *t* with the highest *NPV* (ideally 1). This methodology allows accepting annotations with scores above the upper threshold, reject all below the lower threshold and encourages manual curation of entries with scores in between.

However, incorrect automatic annotations of the sample retrieved for the standard of truth, with very high or very low scores, may impair this methodology, by requiring the annotation of a large number of entries. Hence, it should be allowed to relax *precision* and *NPV* thresholds to values below 1, so that the manual curation efforts are not so demanding. For instance, lowering *precision* will increase the number of annotations automatically accepted. Likewise, lowering *NPV* increases the number of annotations automatically rejected. Therefore, relaxing these metrics introduces a new trade-off that, though accepting erroneous annotations, increases the number of records automatically annotated/rejected, decreasing curation efforts.

An example of the steps required to implement *SamPler*, is presented in Tables S.2-S.4 of the Additional file 2. As shown in Table S.2 of the Additional file 2, a sample of 50 (*x*1) genes, roughly 5% of the potentially metabolic genome, was manually curated to determine whether such genes encoded enzymes and which EC numbers should be assigned to them, thus becoming the standard of truth for such genes.

The automatic annotations for nine (0.1– 0.9) different α values (*x*2) were then calculated and compared with the standard of truth in the array (*x*1, *x*2).

Setting the parameter values to both edges (0.0 and 1.0) may be too extreme, as it may completely eliminate a component of the scorer, thus biasing results. Therefore, combinations with extreme values for the parameters should be carefully considered.

Next, the confusion matrices for each pair (*t*, *x*2) were computed, as shown in Table S.3 of the Additional file 2. For instance, when calculating the confusion matrix for the pair (*t*, *x*2) = (0.5, 0.2), all correct annotations with scores equal or above 0.5 are considered correct (29) and inserted in the CA cell. Every correct annotation below 0.5 (12) was inserted in the IR cell. Likewise, all incorrect *merlin* assignments with scores above 0.5 (4) are inserted in the IA, whereas wrong annotations below the threshold (5) are included in the CR cells. This process is repeated until confusion matrices of all (*t*, *x*2) pairs are calculated. Worth mentioning is the fact that the example presented herein only depicts analysis for *merlin*’s α value, and upper and lower thresholds, to present a clear and concise demonstration.

Finally, as shown in Table S.4 of the Additional file 2, these matrices allow calculating *accuracy*, *precision* and *NPV*. *merlin*’s annotation algorithm highest mean accuracy is associated with *α* = 0.1. For this *α*, all annotations with *t* above 0.9 have a *precision* of 1, which means that *merlin*’s algorithm is correct 100% of the times. Likewise, annotations with *t* below 0.2 have a *NPV* of 1, which means that *merlin* correctly rejects these annotations 100% of the times.

As shown in Table S.5 of the Additional file 2, the user will have to curate manually 301 genes, which represent 30% of the genes that potentially encode enzymes. Often, the *α* with the highest accuracy is simultaneously the one with the highest number of entities to be manually verified. Thus, a curation ratio score, which compromises accuracy with the percentage of records to be curated, is also calculated, according to Eq. 4.

4 $$ curation\ ratio\ score=\frac{accuracy}{\% entries\ to\  be\  curated} $$

This allows a trade-off between of accuracy and curation efforts. Moreover, *merlin* allows users to accept lower *precision* and/or *NPV* (to at least 75%) to decrease the number of entities that will be curated, thus consenting errors in the automatic annotation.

### *SamPler* evaluation

*SamPler* was used to calculate the parameters for several organisms. Four complete genome sequences, for *Lactobacillus rhamnosus* (taxonomy identifier: 568703), *Streptococcus thermophilus* (taxonomy identifier: 322159), *Lactobacillus helveticus* (taxonomy identifier: 326425) and *Nitrosomonas europaea* (taxonomy identifier: 228410), were retrieved from the National Center for Biotechnology Information (NCBI) database. These organisms are of interest for the host group and are being studied in different projects.

*S. thermophilus*, *L. helveticus* and *Lactobacillus rhamnosus* belong to the lactic acid bacteria group (*Lactobacillales* Order), thus having a considerable number of strains and taxonomically close microorganisms, available in the UniProtKB database, whereas, *N. europaea* is the only sequenced strain for this organism, with a relatively low number of taxonomically close microorganisms available in UniProtKB.

**Fig. 3:**
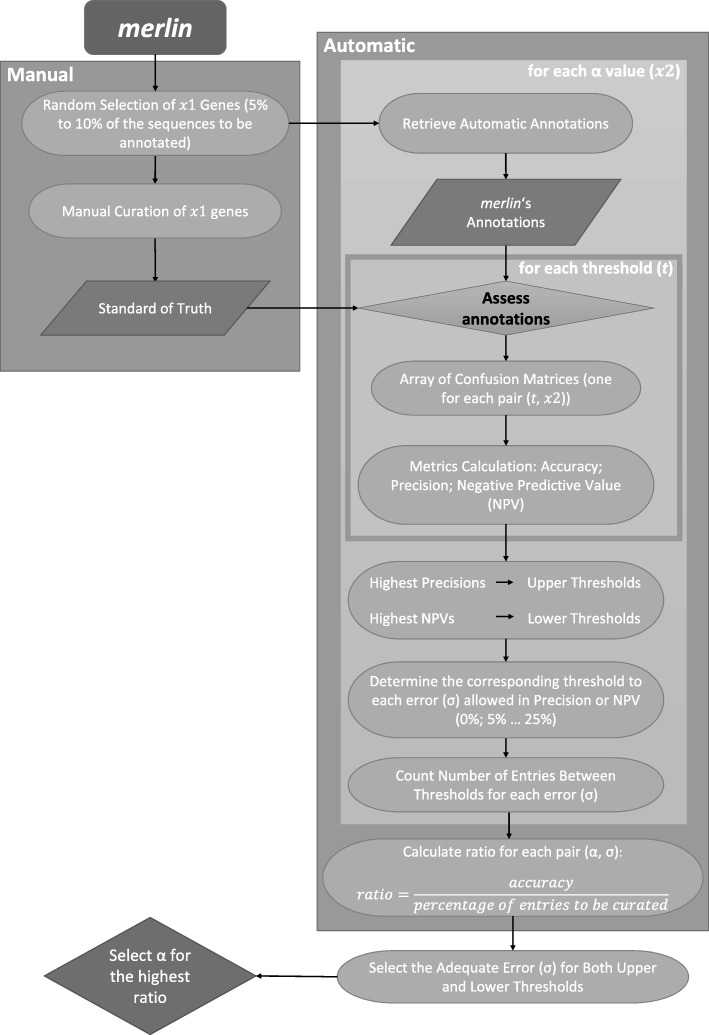
Scheme of the semi-automated method implemented in *merlin*’s EC number annotation tool. *Merlin* proposes × 1 entries (5 to 10% of the sequences to be annotated) for manual curation which will become standard of truth. Then, for each α value the corresponding automatic annotations are retrieved and assessed against the standard of truth. After assessing each entry for each α value, *merlin* calculates a confusion matrix for each pair (threshold, α value). This multi-dimensional array, of confusion matrices, allows calculating the accuracy of each α, and the precision and NPV of each pair. Finally, the number of records between thresholds (taking into account the error allowed in *precision* and *NPV*) are assigned as entries to be curated and the curation ratio score helps determining the best α value and thresholds as a function of the highest accuracy divided by the ratio of entries to be curated. An error up to 25% is allowed in both *precision* and *NPV*

After integrating *SamPler*’s plugin in *merlin*, an automatic genome functional annotation was performed for each genome using the BLAST algorithm against the UniProtKB. A random sample of genes was then automatically collected by *SamPler*. These entries were manually curated inside *merlin*’s environment, following the manual curation scheme in the Additional file 1. Finally, the best parameters settings were calculated by *SamPler*, for both *precision* and *NPV* of 100%. An illustration of the *SamPler* workflow implemented in *merlin* for selecting the best parameters of the annotation algorithm is shown in Fig. 3.

Furthermore, besides assessing the best parameters for the annotation algorithm of the above mentioned organisms, another organism’s annotation (*L. rhamnosus*) was analyzed in detail. In this case, the annotation was performed using initially UniProt/SwissProt as the BLAST database and later, for records without homology hits, against UniProtKB. However, in this case, all EC number annotations were reviewed with the curation workflow (described in Additional file 1), which allows assessing *SamPler*’s calculations and predictions.

## Results and discussion

*SamPler* allows determining the best settings for the parameters of genome functional annotation algorithms, hereby contributing to the improvement of annotations, even when based on biased and redundant data. The manual annotation of a random sample of genes together with the computation of confusion matrices, decreases the manual curation efforts, often required after submitting data to automatic procedures.

*SamPler* was implemented as a plugin for *merlin*’s enzymes annotation algorithm, allowing to select the best parameter values along with the thresholds that determine the number of protein coding genes to be manually annotated.

The analysis performed with *L. rhamnosus* was used to validate *SamPler*’s methodology. All enzymatic genes were manually annotated using the workflow shown in Additional file 1. *SamPler* was used to calculate parameters for two sets of genes, namely genes annotated against UniProt/SwissProt and genes annotated with UniProtKB.

**Fig. 4:**
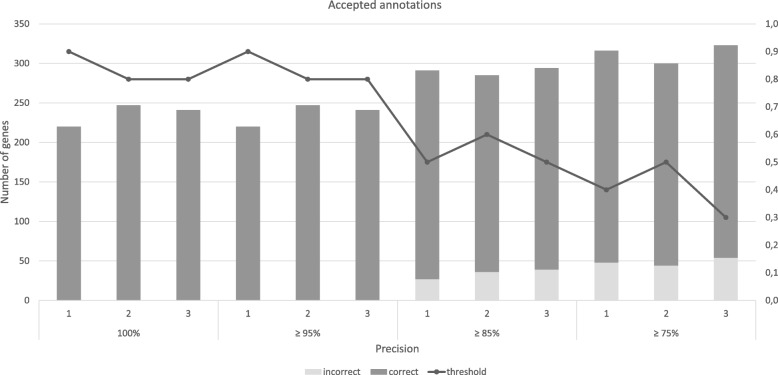
Analysis of the number of genes automatically annotated by merlin, according to SamPler’s proposals for the α parameter and upper threshold. Results shown for genes annotated against UniProt/SwissProt, using 42 genes as standard of truth, for 100, 95, 85 and 75% precision

**Fig. 5:**
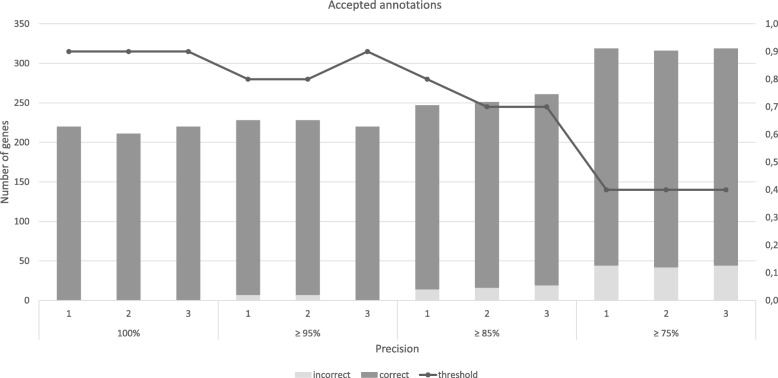
Analysis of the number of genes automatically annotated by merlin, according to SamPler’s proposals for the α parameter and upper threshold. Results shown for genes annotated against UniProt/SwissProt, using 75 genes as standard of truth, for 100, 95, 85 and 75% precision

As shown in Figs. 4, 5, Tables S.7 and S.8 of the Additional file 2, these annotations allowed assessing *precision* and *NPV* to sample size and type of database used for annotation (curated, non-curated and merging of both). Hence, these analyses were performed for records annotated against UniProt/SwissProt (334 genes), UniProtKB (973) and both (1307), for two sample sizes in triplicate (three manually curated samples). The annotation performed with the UniProt/SwissProt provided few records with lower scores. Therefore, to balance the distribution of scores in the sample, the sizes were 42 and 75. For the same reasons, the larger sample size for UniProtKB had 98 entries, whereas, for latter assessment, the sample sizes were 50 and 100.

As expected, generally, lowering the acceptable *precision* and *NPV* allows annotating more records, though accepting errors in such annotations (Tables S.6, S.7 and S.8 of the Additional file 2).

### SwissProt results

When using 42 genes to assess the parameters, lowering the acceptable overall *precision* to 95% will not increase the average number of automatically annotated entries, thus also not adding errors to the annotation. However, selecting the parameters to automatically accept annotations with an overall 85% precision will automatically annotate 290 ± 5 more entries, though 34 ± 6 of these are incorrectly annotated. Yet, in this case, the mean overall effective precision will be ≈88 %  ± 2%. Likewise, when configuring *SamPler* to select parameters that provide an overall annotation *precision* over 75%, 313 ± 12 entries are automatically annotated, though 49 ± 5 of these are wrong, thus the overall effective precision is ≈84 %  ± 1%.

As shown in Table S.6 of the Additional file 2, using 75 genes to determine the standard of truth, provides roughly the same results. Accepting an overall precision of 95% automatically includes 225 ± 5 genes in the annotation, being 5 ± 4 incorrect. For an overall precision of 85 % , 253 ± 7 genes are automatically annotated, 16 ± 3 of which are incorrect. Finally, lowering the acceptable *precision* to 75% will automatically annotate (318 ± 2) genes, from which 43 ± 1 will be wrongly annotated. The effective *precisions* would be ≈98 %  ± 2, ≈ 94 %  ± 1 and ≈86 %  ± 1 respectively. For both sample sizes, the *α* and the upper threshold values tend to go down, when accepting lower *precisions*.

When analyzing annotations obtained with this database, for both sample sizes in all replicates, lowering the overall *NPV* does not affect results, hence no incorrect rejections are performed in the annotation. Likewise, the lower threshold and *α* remain stable for all acceptable *NPV*.

### UniprotKB results

The analyses performed for entries annotated against UniProtKB provided overall results similar to the previous ones. Again, as shown in Table S.7 of the Additional file 2, the effective *precision* is always within the range accepted by *SamPler*.

Configuring *SamPler* to provide an overall *precision* of 95%, for the 50 genes’ sample, does not misannotate any genes, thus providing an effective *precision* of 100%. Furthermore, when the algorithm is configured to accept 15% of error in *precision*, only 7 ±7 incorrect entries, within 188 ± 10, are automatically accepted as correct (*precision* = 96 %  ± 4). Finally, *SamPler* has a *precision* of 89 %  ± 4, which corresponds to 23 ± 10 misannotations for 211 ± 13 automatically annotated records, when calculating the parameters for a minimum *precision* of 75% in the sample’s annotations.

Regarding the analysis of the results obtained with the larger sample (98 genes), accepting 5% of error in the *precision* of the annotation, provides similar results to the default 100 % *precision*. In this case, the mean of the automatically annotated genes is 166 ± 9, with 1 ± 2 misannotations, instead of 162 ± 4. Again, decreasing the acceptable *precision*, allows automatically annotating more entries, without significant errors 193 ± 3 (6 ± 2 misannotations) and 229 ± 33 (36 ± 24 misannotations), for 85% and 75 % *precision* respectively. For annotations in UniProtKB, though the threshold decreases along with *precision*, the mean of the α parameter remains fairly constant (average of 0.73 − 0.8 for the smaller sample and 0.63 − 0.77 for the larger sample).

Analyzes of the automatically rejected entries in UniProtKB provided interesting results. Most annotations with low scores corresponded to incorrect annotations, thus the proposed lower threshold rejected hundreds of annotations. In fact, for a special case (gene CAR87684.1), according to the workflow, the annotation proposed by *merlin* was correct, though with a very small score (0.12). Hence, whenever this gene was present in the sample, the *NPV* would be significantly affected. Nevertheless, this effect can be softened by accepting a 5% error in *NPV*, as shown in Table S.7 of Additional file 2, although this was the only overall *NPV* for which the mean of the effective *NPV* ’s was below the acceptable, for both sample sizes. Overall, the average *α* varied between (0.73 and 0.9) for both samples. As expected, the lower threshold tended to increase with the decrease of the *NPV*.

### Merged databases

Performing these analyzes for all entries annotated with both homology searches, provides results similar to the entries annotated against UniProtKB, as ≈73% of the annotations were obtained from that database. As expected, the mean of the effective *precision* is above the minimum limits configured in *SamPler*, for all intervals in all samples. Regarding the *NPV*, results are also very good, although when configuring *SamPler* to provide an overall *NPV* of 85% or 95%, analyses show that the means of the effective *NPV* are slightly below the limit, for both samples.

Also, the upper threshold decreases together with *precision* and the lower threshold increases when lowering the *NPV*. The *α* parameter values are fairly constant for both samples and metrics, though (for a *precision* of 100%) in sample 50.1 the calculated *α* is low when compared to samples 50.2 and 50.3.

### Other organisms’ results

Regarding *S. thermophilus*, *merlin*’s *SamPler* suggested an α value of 0.4 (accuracy of 0.691) with a curation ratio score of 1.8 (Table 1), and the manual curation of 331 entries (38% of the number of potentially enzymatic genes), when setting the lower and upper thresholds to 0.2 to 0.6, respectively. As shown in Table S.9 of the Additional file 2, three α values, viz. 0.1, 0.3 and 0.7, had a higher calculated accuracy (0.696), but the number of entries to be curated would be 40%, 45% and 46%, respectively. Thus, the increase in accuracy would not justify the extra curation efforts.

**Table 1:** Main results of implementing the semi-automated method in merlin’s EC number annotation tool using three complete protein sequences as test cases, one of each single-different organism

Organism	*S. termophilus*	*L. helveticus*	*N. europaea*
α	0.4	0.7	0.2
Upper Threshold	0.6	0.8	0.8
Lower Threshold	0.2	0.3	0.1
Genes to Be Curated	331	264	624
% for curation	38%	36%	56%
Accuracy	0.691	0.753	0.591
Curation ratio score	1.8	2.11	1.05
Sample size	50 (5.8%)	50 (6.7%)	60 (5.4%)
Potentially metabolic	862	741	1114
Genome	1716	1685	2462

Regarding *L. helveticus*, *merlin*’s *SamPler* proposed an α value of 0.7, with a curation ratio score of 2.11 (Table 1), and the manual curation of 264 entries (36% of the number of potentially enzymatic genes), when setting the lower and upper thresholds to 0.3 and 0.8, respectively. For this organism, as shown in Table S.10 of the Additional file 2, only one α value (0.9) had a higher accuracy (0.758), but it required the curation of 45% of the annotations, that is 67 more entries than for *α* = 0.7.

Finally, for *N. europaea, SamPler*’s results are substantially different from the previous. The proposed α was 0.2 and the thresholds were 0.8 and 0.1, for upper and lower thresholds, respectively. Recall that this organism has few closely related *species* available in the BLAST database. If α = 0.1 was selected, instead of the current 624, 693 records would have to be curated (Table S.11 of the Additional file 2).

The complete calculation reports, provided by *merlin*’s *SamPler*, are available in Tables S.9 to S.11 of the Additional file 2, for *S. thermophilus*, *L. helveticus* and *N. europaea*, respectively.

Indeed, the results provided by *SamPler* are correlated with the organisms’ taxonomic families’ presence in the BLAST databases. For instance, the recommendation of *α*_*sth*_ = 0.4 for *S. thermophilus* or *α*_*lhe*_ = 0.7 for *L. helveticus*, can be associated with the fact that these microorganisms have multiple strains and an even higher number of closely related microorganisms (surprisingly from the same *genus*) with complete genome functional annotations available in UniProtKB. Hence, both the frequency of the EC number and the taxonomy of the homologous genes annotated with such EC numbers should be taken into account when calculating the annotation score.

On the other hand, *N. europaea* is poorly described in UniProtKB. Hence, it was expected that the selected *α* (0.2) would enhance the taxonomy component in the final score ( *α*_*neu*_ = 0.2 *x score*_*frequency*_ + 0.8 *score*_*taxonomy*_). The absence of taxonomically close, well characterized, organisms increases the relevance of the few related records, enhancing the taxonomy score. Notice that *N. europaea* also presents the highest percentage of genes that should be manually curated.

## Conclusions

Here *SamPler*, a tool aimed at improving genome functional annotations, is showcased. The results of this work show that the parametrization of *merlin*’s enzyme annotation is not straightforward. In fact, the selected *α* values for two Lactobacillales were 0.4 and 0.7, for *S. thermophilus* and *L. helveticus*, respectively. Still, for *N. europaea*’s the *α* parameter value was 0.2. The automatic annotation/rejection thresholds also varied significantly among the projects. These results show that each project is unique, with several factors influencing the outcome of the annotation, such as the availability of manually curated or incorrect annotations for the organism’s genes.

Analyses of the complete curation of *L. rhamnosus* show that using non-curated databases to perform annotations provide very few correct annotations when compared with the curated ones. Also, the effective errors when allowing values both for *precision* and *NPV* below 100%, are mostly within acceptable ranges. Therefore, the results of this work demonstrate that larger projects can benefit from lower *precision* and *NPV* thresholds, as these may decrease the curation efforts, while incorrectly annotating (or rejecting) very few entries.

Hence, performing high-quality semi-automatic genome functional annotations should involve systematic and reproducible methodologies, that reduce both human error and data bias, such as the *SamPler* presented in this work. Indeed, *SamPler* accepted and rejected a significant number of entries for all organisms while proposing a well-aimed number of genes that should be manually curated, clearly improving, guiding and hastening the manual curation process.

## Additional files

Additional file 1: Example of a manual curation workflow for the genome functional annotation of the microorganism *Lactobacillus rhamnosus* using merlin. (PDF 173 kb)

Additional file 2: SamPler demonstration. Results obtained after applying SamPler procedure to 4 genome functional annotations. (XLSX 65 kb)

## Data Availability

*merlin is* freely available at http://www.merlin-sysbio.org/. *SamPler* is available at https://gitlab.bio.di.uminho.pt/merlin-sysbio/merlin-sampler. The data generated or analysed during this study are included in this published article and its supplementary information files. The genome functional annotation analysed during the current study is available from the corresponding author on reasonable request.

## Abbreviations

BLAST: Basic Local Alignment Search Tool
CA: Correct Annotations
CR: Correct Rejections
EC: Enzyme Commission
GO: Gene Ontology
IA: Incorrect Annotations
IR: Incorrect Rejections
KEGG: Kyoto Encyclopedia of Genes and Genomes
NCBI: National Center for Biotechnology Information
NPV: Negative Predictive Value
TC: Transport Classification
UniProtKB: Universal Protein Resource Knowledgebase
