# Supplementary material for: SamPler – a novel method for selecting parameters for gene functional annotation routines
Source: BMC Bioinformatics. 2019 Sep 5;20:454. doi: 10.1186/s12859-019-3038-4 (PMC6727554; doi:10.1186/s12859-019-3038-4)
Supplement: Supplementary file 1 — Example of a manual curation workflow for the genome functional annotation of the microorganism Lactobacillus rhamnosus using merlin. (PDF 173 kb) [file 12859_2019_3038_MOESM1_ESM.pdf]

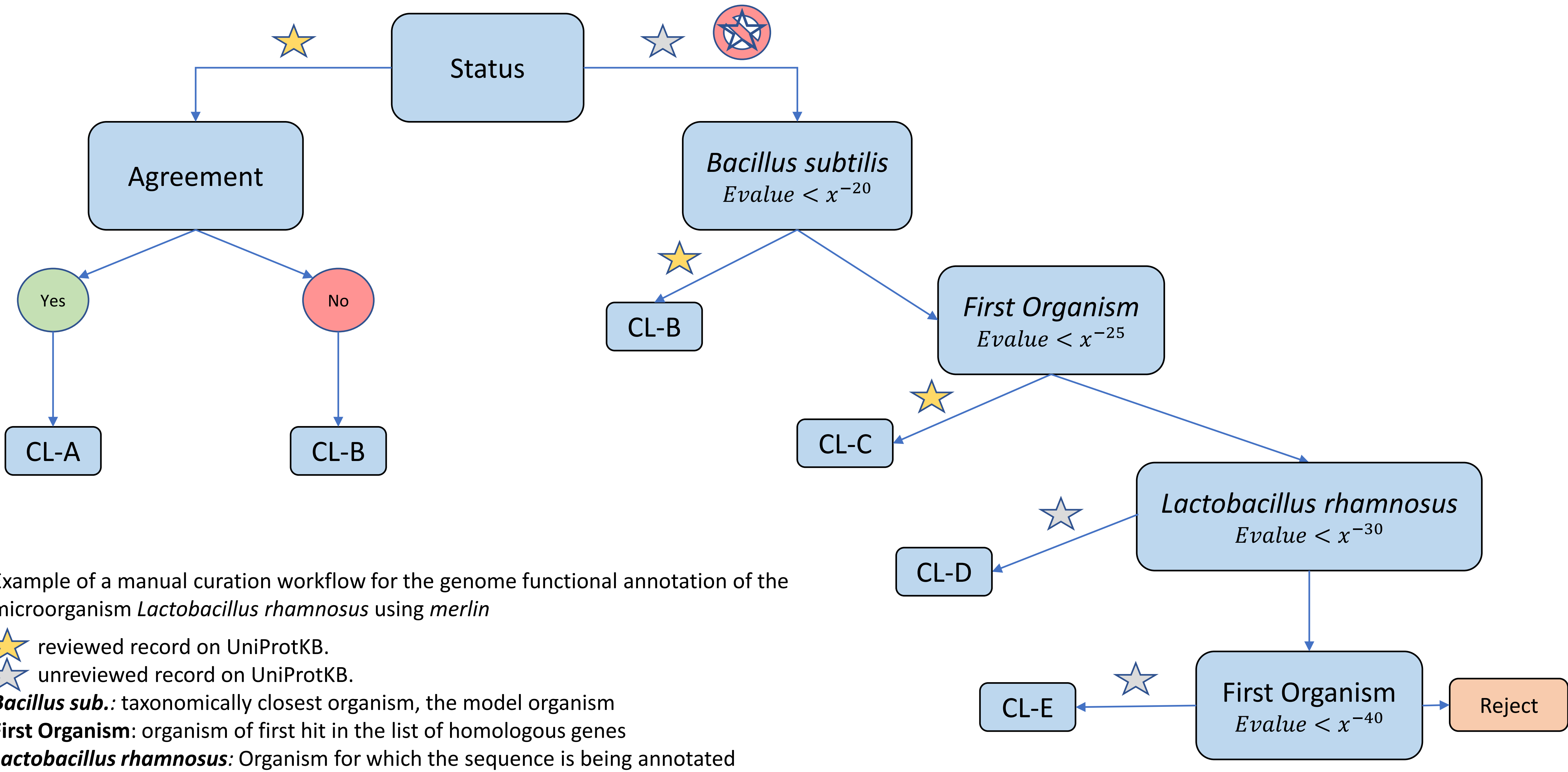

Example of a manual curation workflow for the genome functional annotation of the microorganism *Lactobacillus rhamnosus* using *merlin*

★ reviewed record on UniProtKB.
☆ unreviewed record on UniProtKB.
Bacillus sub.: taxonomically closest organism, the model organism
First Organism: organism of first hit in the list of homologous genes
Lactobacillus rhamnosus: Organism for which the sequence is being annotated
CL-A/B/C/D/E: Annotation Confidence Levels. A, B and C stands for Very High, High and Medium Confidences, while D and E stands for Low and Very Low Confidences, respectively.
